# Supplementary material for: Clinical outcomes of endoscopic submucosal dissection for colorectal neoplasms: A single-center experience in Southern Taiwan
Source: PLoS One. 2022 Oct 7;17(10):e0275723. doi: 10.1371/journal.pone.0275723 (PMC9543958; doi:10.1371/journal.pone.0275723)
Supplement: Attachment — Submitted filename: Response to Reviewers.docx [file pone.0275723.s001.docx]

For Editor Comments

1. As the study aimed to study ESD outcome in Southern Taiwan, please provide short discussion for their result with other reports of colonic resection from the same area with similar background of ESD development

Ans: We provided in Page 17, Line 221 to 228

As for the result of colorectal ESD in the same area with a similar background, Choo et al. revealed en-bloc resection rate (72.7%) and R0 resection rate (66.7%) with perforation rate (15.2%) when the ESD technique was newly developed in Southern Taiwan [18]. A study by Tseng et al. showed en-bloc resection rate (90.2%) and R0 resection rate (89.1%) with perforation rate (12%) [19]. Although the studies mentioned above vary, improvements in en-bloc resection rate, R0 resection rate, and complication rate were observed in our study, contributing to the accumulation of ESD experience and improvement of ESD training programs in Southern Taiwanese hospitals.

For Reviewer #1:

1. The method of EUS (mini-probe or radial /linear scope).

Ans: We provided in Page 6, Line 98 to 99

Our EUS procedures used a miniature Probe (UM-2R; Olympus Medical Systems, Tokyo, Japan) and an ultrasound system (EU-ME2 Premier Plus; Olympus Medical Systems, Tokyo, Japan).

2. They should clearly mention that how many cases had endoscopic procedure before ESD (EMR or tried for polypectomy in another center)

Ans: We provided in Page 5, Line 74 to 75

None of these patients received previous EMR or polypectomy. Sixteen cases underwent biopsy only, which were unrelated to submucosal fibrosis during ESD.

For Reviewer #2:

1. “Clinical outcomes of colon endoscopic submucosal dissection for colonic neoplasms: A single center experience in South Taiwan” => Revise as “Clinical outcomes of endoscopic submucosal dissection for colorectal neoplasms: A single center experience in Southern Taiwan.”

Ans: We already revised in Page 1, Line 1 to 3.

2. Please shorten the background and study aim in the abstract part.

Ans: Already shortened as Page 2, Row 21 to 24.

3. Please revise “colonic” neoplasm as “colorectal” neoplasm in the title, abstract and manuscript given that rectal neoplasms have been included as well. (For example, “colonic and rectal lesions” could be revised as “colorectal lesions” in the abstract part.)

Ans: Already revised and highlighted in file: Revised Manuscript with Track Changes

4. Please specify the “percentage” of patients with complications in the abstract part.

Ans: Already Specified in Page 2, Line 31 to 33.

5. How to define “minor” perforation?

Ans: Mentioned in Page 9, Line 140 to 142.

Our minimal perforation was defined as muscle layer defect without observation of mesenteric fat or intra-peritoneum organ and pneumoperitoneum.

6. Please use serrated sessile “lesions” rather than “polyps” in the manuscript and tables.

Ans: Already revised and highlighted in file: Revised Manuscript with Track Changes

7. In “Table 2”, please specify the percentage of HGD/LGD lesions.

Ans: Already revised and highlighted in file: Revised Manuscript with Track Changes

8. Please explain why using ESD for lesions less than 20mm. According to recommendations from international societies, colorectal LST which are less than 20mm could be managed by EMR.

Ans: Explained in Page 5, Line 78 to 81.

We chose ESD over EMR in some colorectal lateral spreading tumors less than 20 mm in cases of suspected lesions with limited submucosal invasion or difficult locations for en-bloc EMR, such as ileocecal valve, hepatic/splenic flexure, and sigmoid colon.

9. Please define “long ESD time” in the Materials and Methods part rather than in the Result part.

Ans: Revised in Page 7 to 8, Line 120 to 122.

10. Was submucosal fibrosis associated with long ESD time?

Ans: Mentioned in Page 19, Line 260-262

Although a study in Japan revealed fibrosis as a factor influencing long procedure times, there was no statistical significance in our study due to the limited cases of submucosal fibrosis.

11. What was the proficiency of endoscopists in EUS? What kind the EUS used in this study (miniature probe with same frequency? radial echoendoscope?)?

Ans: We provided in Page 6, Line 96 to 99

EUS procedures were performed by two experienced endoscopists who have performed more than 2000 EUS procedures. Our EUS procedures used a miniature Probe (UM-2R; Olympus Medical Systems, Tokyo, Japan) and an ultrasound system (EU-ME2 Premier Plus; Olympus Medical Systems, Tokyo, Japan).

12. Please evaluate inter-observer and intra-observer variation for EUS interpretation.

Ans: We selected those six cases of discordance to evaluate inter-observer and intra-observer variation.

|  | Previous EUS interpretation of depth invasion | EUS Interpretation in 2022  (Intra-observer) | Another EUS endoscopist’s interpretation in 2022  (Inter-observer) |
| --- | --- | --- | --- |
| Case 1 | Submucosal | Submucosal | Submucosal |
| Case 2 | Submucosal | Submucosal | Submucosal |
| Case 3 | Mucosal | Mucosal | Mucosal |
| Case 4 | Submucosal | Submucosal | Submucosal |
| Case 5 | Mucosal | Mucosal | Submucosal |
| Case 6 | Submucosal | Submucosal | Submucosal |

13. Please send manuscript for English language editing.

Ans: Already done and the revision was highlighted in file: Revised Manuscript with Track Changes
