# Supplementary material for: Clinical outcomes of endoscopic submucosal dissection for colorectal neoplasms: A single-center experience in Southern Taiwan
Source: PLoS One. 2022 Oct 7;17(10):e0275723. doi: 10.1371/journal.pone.0275723 (PMC9543958; doi:10.1371/journal.pone.0275723)
Supplement: Attachment — Submitted filename: Response to reviewer (minor revision).docx [file pone.0275723.s002.docx]

For Reviewer #2:

1) Please use pathological findings as standard reference and calculate the interobserver and intra-observer variation which presented as kappa value.

Ans:

We used SPSS to calculate Kappa value.

Intra-observer (Previous interpretation and interpretation in 2022): Kappa value: 1

Inter-observer (Previous interpretation and interpretation by another EUS endoscopist in 2022): Kappa value: 0.897

2) Please provide a cleaner version of manuscript with language editing parts deleted.

Ans: I had provided two versions of manuscript; one is clean version (File name: manuscript) and another one is track-change version (File name: Revised Manuscript with Track Changes).
